# The Silent Weight of Nutrition Data in Glucagon-Like Peptide-1 Receptor Agonists Clinical Trials

**DOI:** 10.1016/j.advnut.2025.100498

**Published:** 2025-08-16

**Authors:** Hassan S Dashti, Lukasz Szczerbinski

**Affiliations:** 1 Department of Anesthesia, Critical Care and Pain Medicine, Massachusetts General Hospital, Boston, MA, United States; 2 Division of Nutrition, Harvard Medical School, Boston, MA, United States; 3 Division of Sleep Medicine, Harvard Medical School, Boston, MA, United States; 4 Center for Genomic Medicine, Massachusetts General Hospital, Boston, MA, United States; 5 Diabetes Unit, Department of Medicine, Massachusetts General Hospital, Boston, MA, United States; 6 Programs in Metabolism and Medical & Population Genetics, Broad Institute, Cambridge, MA, United States; 7 Department of Endocrinology, Diabetology and Internal Medicine, Medical University of Bialystok, Bialystok, Poland; 8 Clinical Research Centre, Medical University of Bialystok, Bialystok, Poland

The recent introduction of glucagon-like peptide-1 receptor agonists (GLP-1RAs) into obesity management represents one of the most notable pharmacological advances in recent decades. These medications, particularly semaglutide and tirzepatide, are reshaping obesity treatment by producing substantial weight loss, primarily through appetite suppression and reduced food intake [1]. Yet an important question remains: How do these medications influence dietary intake, the foundational behavioral cornerstone of obesity management?

In their comprehensive scoping review, Babazadeh et al. examined 129 clinical trials evaluating GLP-1RA efficacy published through December 2024 and found that most studies neglected to assess or report data on dietary intake or eating behaviors [5]. Although 57 of 129 investigated trials (∼44%) reported some form of “lifestyle counseling” along with GLP-1RA, only 36 (28%) included dietary assessment, and just 10 (8%) reported on dietary intake. Even among these 10 studies, dietary assessment methods were often limited to single-point ad libitum meal tests, which are poorly suited to capture meaningful, real-world dietary changes during GLP-1RA therapy. Trials that used more comprehensive assessment tools (e.g., food diaries, 24-hour recalls) were few, underpowered, and focused primarily on older GLP-1RA agents such as liraglutide. Notably, for semaglutide, the current market leader, only 2 trials reported dietary intake data.

The omission of dietary data in GLP-1RA clinical trials represents a key shortcoming in the evidence base that limits our ability to fully understand the therapeutic impact, real-world applicability, and long-term sustainability of these medications. Without dietary intake data, it remains unclear whether the observed weight loss reported in the trials was achieved solely through pharmacologic mechanisms or through synergistic behavioral adaptations, particularly in trials that included lifestyle counseling. The nature of any nutritional adaptation, including changes in dietary quantity, quality, or eating behaviors, remains largely unknown. The lack of dietary data also prevents evaluation of whether the substantial reductions in appetite and energy intake associated with GLP-1RAs contribute to malnutrition and deficiencies in essential nutrients like iron, calcium, magnesium, zinc, or vitamin D [2]. This concern is compounded by common gastrointestinal side effects (e.g., nausea, diarrhea, and vomiting) that can influence food choices and impair nutrient absorption. In addition, risks of low protein intake and consequent loss of muscle and bone mass cannot be adequately assessed. It is also unclear whether changes in food cravings or taste preferences during treatment undermine adherence to dietary recommendations for fruits and vegetables, whole grains, legumes, and other nutrient-dense foods. Validated dietary assessments have long been integrated into large cardiometabolic trials. For example, studies in diabetes prevention have routinely collected and reported detailed dietary data (for example, [3]). The absence of such measures in many GLP-1RA trials reflects a concerning de-emphasis on nutrition in recent pharmacologic obesity research.

The review by Babazadeh et al. also highlights the limited and inconsistent reporting of “lifestyle counseling” in GLP-1RA clinical trials that included various forms of behavioral interventions [5]. Some studies specified the involvement of qualified healthcare professionals, such as behavioral therapists or registered dietitian nutritionists, and there was considerable variability and a general lack of detail regarding the content, delivery method, and frequency of these behavioral interventions. A recent joint advisory from leading nutrition and obesity societies [4] emphasizes the importance of integrating structured, evidence-based lifestyle interventions alongside pharmacologic treatment. This includes comprehensive dietary counseling, physical activity guidance, and behavioral support to enhance efficacy, mitigate side effects, and promote long-term weight maintenance. The advisory argues that nutrition should be a foundational element of care, not a secondary add-on. Yet the absence of such detail in the published GLP-1RA literature makes it impossible to determine how behavioral components may have contributed to reported outcomes. As GLP-1RA prescribing continues to accelerate, often in primary care settings with limited nutrition guidance, the lack of clear reporting on behavioral interventions, coupled with the limited training in behavior change counseling among physicians, undermines the translation of trial results into real-world effectiveness.

Moving forward, GLP-1RA clinical trials must incorporate repeated dietary assessments using validated dietary intake tools, and should clearly report who delivers behavioral interventions and how, and define lifestyle protocols with the same rigor applied to other aspects of the trial design. Nutrition and behavioral components should not be treated as secondary. When dietary data are collected (as some trial registrations suggest), they should be reported comprehensively, beginning with baseline habitual intake. This includes reporting of total calorie intake, dietary quality (e.g., consumption of fruits, vegetables, and ultraprocessed foods), meal timing (e.g., overnight fasting, meal and snack distribution and frequency [especially in light of gastrointestinal side effects that may alter eating patterns]), food preferences and cravings (e.g., for sugary or high-fat foods), and other lifestyle behaviors such as sleep and physical activity. As emphasized by the recent joint advisory [4], clinicians urgently need GLP-1RA–specific dietary guidance informed directly by trial data rather than extrapolated from bariatric surgery or low-calorie weight loss trials.

Ultimately, Babazadeh et al. highlights what is missing. Without robust data on dietary intake and eating behavior, the current evidence base leaves key questions about the full impact of GLP-1RAs unanswered. Until such measures become standard, we risk overlooking key opportunities to inform comprehensive, evidence-based, patient-centered obesity care.

## Author contributions

Both authors read and approved the final manuscript.

## Funding

This work was supported by the National Institutes of Health (grant number R00HL153795 to HSD).

## Conflict of Interest

HSD and LS report a relationship with Eli Lilly and Company that includes: consulting or advisory. HSD is an Associate Editor for *Advances in Nutrition*.
